# Ab-origin: an enhanced tool to identify the sourcing gene segments in germline for rearranged antibodies

**DOI:** 10.1186/1471-2105-9-S12-S20

**Published:** 2008-12-12

**Authors:** Xiaojing Wang, Di Wu, Siyuan Zheng, Jing Sun, Lin Tao, Yixue Li, Zhiwei Cao

**Affiliations:** 1 Bioinformatics Center, Key Lab of Systems Biology, Shanghai Institutes for Biological Sciences, Chinese Academy of Sciences; Graduate School of the Chinese Academy of Sciences, 320 YueYang Road, Shanghai 200031, PR China; 2 Shanghai Center for Bioinformation Technology, 100 Qinzhou Road, Shanghai, 200235, PR China; 3 College of Life science and Biotechnology, Tongji University, Shanghai, 200092, PR China

## Abstract

**Background:**

In the adaptive immune system, variable regions of immunoglobulin (IG) are encoded by random recombination of variable (V), diversity (D), and joining (J) gene segments in the germline. Partitioning the functional antibody sequences to their sourcing germline gene segments is vital not only for understanding antibody maturation but also for promoting the potential engineering of the therapeutic antibodies. To date, several tools have been developed to perform such "trace-back" calculations. Yet, the predicting ability and processing volume of those tools vary significantly for different sets of data. Moreover, none of them give a confidence for immunoglobulin heavy diversity (IGHD) identification. Developing fast, efficient and enhanced tools is always needed with the booming of immunological data.

**Results:**

Here, a program named Ab-origin is presented. It is designed by batch query against germline databases based on empirical knowledge, optimized scoring scheme and appropriate parameters. Special efforts have been paid to improve the identification accuracy of the short and volatile region, IGHD. In particular, a threshold score for certain sensitivity and specificity is provided to give the confidence level of the IGHD identification.

**Conclusion:**

When evaluated using different sets of both simulated data and experimental data, Ab-origin outperformed all the other five popular tools in terms of prediction accuracy. The features of batch query and confidence indication of IGHD identification would provide extra help to users. The program is freely available at .

## Background

One of the strategies our immune system adopts to fight off intruders is to produce appropriate antibodies to recognize and neutralize foreign molecules specifically. This flexibility and robustness of adaptive immune system is mainly achieved by almost unlimited antibody diversity. As a homodimer of heavy and light peptide chains, each antibody contains a unique variable region encoded by variable (V), diversity (D) and joining (J) gene fragments (V and J segments only in the case of light chain) [1,2]. These variable regions play a predominant role in determining the antibody specificity. In contrast to the potentially countless different antigens from the environment, the total sets of gene segments responsible for encoding are highly limited at the genome level. For instance, it has been found that the numbers of gene segments encoding heavy chain in human genome are only about 49 for V, 27 for D and 6 for J segments (from IMGT/GENE-DB). The mechanism by which the diversified antibodies are produced based on limited gene segments has always been a topic of interest in molecular immunology. It is generally believed that the antibody diversity is mainly contributed by rearrangement among gene segments, junctional flexibility, somatic hypermutation and the pair matching between heavy and light chains [3]. In fact, it is only through the V(D)J rearrangement process (the recombining of the pre-existing V, (D), J gene segments) the immune system may theoretically yield 10^4 ^diverse antibody genes for heavy chain (10^2 ^for light chain). In addition, the modifications such as flexible junction [4,5], N-region addition [5] during recombination process and somatic hypermutation during an immune response [6,7], will further lead to considerable increase in diversity and specificity. This process makes every antibody unique, only triggering a high-affinity response to one or one type of antigens.

This complicated process has aroused much interest because abnormal antibodies are often found to relate to serious diseases, such as systemic lupus erythematosus [8-10], multiple sclerosis [11] and rheumatoid arthritis [9]. Thus, analyzing the features and origins of different antibodies would be useful not only to academic researches but also to clinical applications, where partitioning the functional antibody gene to the closest V, D, J gene segments in the germline has become increasingly required. Various tools have been developed to assign rearranged sequences to their germline V, (D) and J counterparts. Some are based on local sequence alignment to find the best match between mature antibody genes and V, (D), and J gene segments, such as DNAPLOT [12], IMGT/V-QUEST [13,14], JOINSOLVER [15] and SoDA [16]. IMGT/V-QUEST is the first automatic tool to analyze immunoglobulin junctional regions and is thus widely applied [13,14]. JOINSOLVER incorporates two relatively conserved motifs, "TAT TAC TGT" and "C TGG GG", to find the margin of complementarity determining region three (CDR3) [15]. Good performance is also achieved by a three-dimensional dynamic programming algorithm for VDJ segments in SoDA [16]. Another group of methods have applied statistical models, such as the hidden markov model (HMM), to obtain the optimized parameters fitting to the rearranged antibody, such as VDJsolver and iHMMune-align [17,18]. Although these type of methods provide alternative ways to locate the best matched gene segments in the germline, model robustness relies heavily on the quality and diversity of training data sets in order to obtain consistently good performance for different varieties of antibodies [17].

For many years, researchers have relied on DNAPLOT and IMGT/VQUEST for immunoglobulin sequence alignment. As different programs have their respective advantages and disadvantages, several approaches have been reported in recent years to suit different needs [15-18]. For instance, JOINSOLVER was developed specifically for analyzing CDR3 regions, which gives best results to sequences without mutations in the two conserved motifs [15]. While SoDA is often used to analyze a small number of sequences with low mutation level [16]. Despite that, none of them give quantitative measures about confidence level, which could be a useful guide to the users especially when identification accuracy is not high enough for IGHD.

In this paper, we describe a fast and efficient tool for general analysis which partitions functional antibody sequences to corresponding gene segments, with substantive refinement of algorithm parameters and more extensive validation based on a preliminary work [19]. In particular, for users' reference, a confidence indicator is provided in terms of the scoring threshold corresponding to certain specificity and sensitivity for IGHD identification.

In our method, the empirical knowledge from clonally unrelated rearranged sequences was incorporated and natural antibody sequences were used to confirm the feasibility of Ab-origin rather than purely simulated sequences. BLAST algorithm [20,21] with customized parameters and window-sliding algorithm were adopted to realize the process. The performance of Ab-origin was evaluated through independent set of simulated antibody sequences, as well as being compared to other five popular tools. Ab-origin was developed using Java language.

## Results

### General information on human IGH germlines

The numbers of non-redundant alleles from IMGT are 267, 32, and 16 for IGHV, IGHD and IGHJ respectively. The statistics showed that the full-length IGHV germline sequences are 295.89 ± 3.38 nt long on average, ranging from 288 to 305 nt, while the data for IGHJ segments is 53.92 ± 6.14 nt, ranging from 48 to 63 nt. Comparing to IGHV and IGHJ, the average length of IGHD sequences is only 24.35 ± 7.13 nt, with much larger variations in length from 11 to 37 nt.

### Choosing optimized scoring scheme for IGHD identification

**Figure 1:**
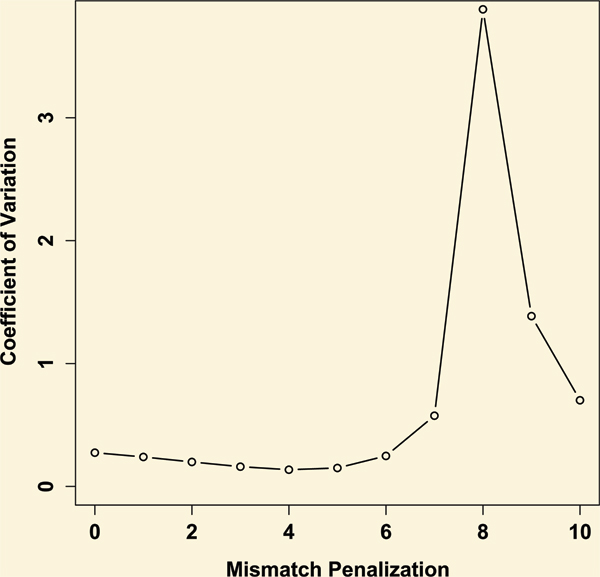
**The influence of different penalty score to the coefficient of variation for alignment scores**. Scoring scheme of +5/-x(x from zero to ten, stepping one) were tested using random simulated sequences of length for 5 to 64. X axis indicates penalty score per mismatch. Y axis represents the coefficients of variation for alignment score. The result shows the +5/-4 scheme has the minimal coefficient of variation.

Based on the analysis of results from Monte Carlo simulation, an optimized scoring scheme is developed to minimize the possible effect resulted from large variation in length of V-to-J region. The results from the simulation have been plotted into Figure 1. It can be seen that the scoring scheme of +5/-4 shows the minimal coefficient of variations under different length of V-to-J region from 5 to 64.

### Initial evaluation through IGHV3-23 sequence set

A set of 6329 rearranged antibody sequences obtained by amplifying the IGHV3-23-IGHD-IGHJ joints by PCR were collected [18], and 500 were randomly selected as input data to evaluate the IGHV identification performance of Ab-origin, together with five other tools (IMGT/V-QUEST, SoDA, JOINSOLVER, VDJsolver and iHMMune-align). As shown in Table 1, the six tools all gave good performance in IGHV3-23 identification. It is evident that, although iHMMune-align was designed to analyze rearrangement with no insertion or deletion within IGHV gene [17], it did not pick up any false positives.

**Table 1:** Results of IGHV identification of 500 sampled sequences using six tools.

	Number of being rejected^a^	Incorrect pickups of IGHV3-23^b^	Correct pickups of IGHV3-23	Accuracy (%)^c^
Ab-origin				
IMGT\V-QUEST	0	1	499	~100
SoDA	1	0	499	~100
JOINSOLVER	1	17	482	96.4*
VDJsolver	0	0	500	100
iHMMune-align	24	0	476	95.2*

The numbers of correct and incorrect pickups are shown here, together with the number of input sequences which can't be deciphered (being rejected) by the six tools. A correct pickup is defined as a successful identification of IGHV3-23 gene within the 500 samples, allowing for mismatched alleles.

* Demonstrate that the accuracy of Ab-origin is significantly higher than that of this tool.

^a ^Number of the input sequences cannot be deciphered by the tool due to various reasons.

^b ^Number of the incorrect pickups among the sequences already been deciphered.

^c ^Accuracy = Correct pickups/500

**Figure 2:**
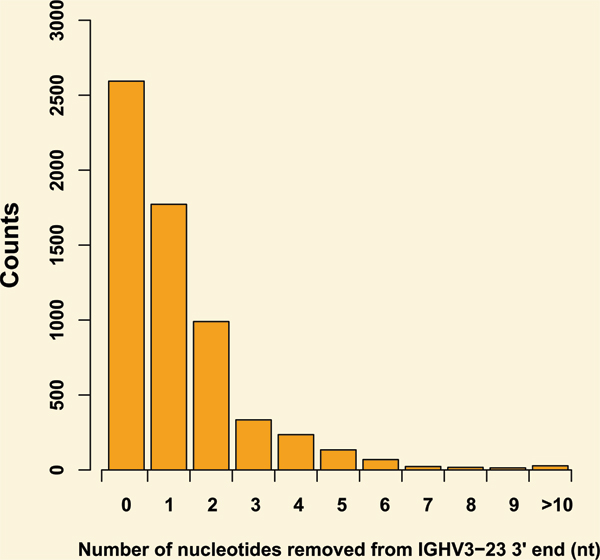
**The distribution of removed nucleotides length from IGHV3-23 3' end**. The length distribution of nucleotides removed from the 3' end of IGHV3-23 during antibody recombination process for 6329 sequences in total.

This set of experimental data could also be applied to derive parameter setting of junctional flexibility. After mapping the rearranged sequences to their original IGHV3-23 gene segments, the number of nucleotides that have been removed from the 3' end of IGHV3-23 during the recombination process can be obtained. Figure 2 shows the frequency distribution of nucleotide number being removed from the 3' end of the 6329 sequences. More than 95% of the junctional flexibility length are equal or smaller than five nucleotide in our study, a result indirectly supported by another study [22], thus the allowed length of junctional flexibility is set to 0–5 nt in our following simulation model.

### Further validation using simulated data set of full-length variable regions for heavy chain

Because of the scarcity of experimentally derived antibody sequences with known germline gene segments, artificial sequences were often generated to validate predicting algorithms [16]. 32000 pieces of antibody sequences of variable regions for heavy chain were initially obtained by simulation described in the Methods section. The length distribution of V-to-J region of these sequences was compared with that of 4450 real antibody sequences from IMGT database (see Additional File 1: Figure S1). With 26.80 ± 8.35 nt (simulated) compared to 26.53 ± 10.26 nt (real), the result indicates that no significant difference in length is observed between simulated and real sequences (*p *> 0.05, t test). Therefore, the simulated sequences are expected to be applicable for further validation. It is noted that different tools often use different germline repertoires which could make a difference when comparing their performance. To ensure fairness, only simulated sequences which include those common germline repertoires among the six tools were retained. During the validation, 1000 pieces of such sequences randomly selected were treated as testing data, the same version of the IMGT germline gene repertoire is recruited as much as possible between all programs. The performances of different programs are summarized into Table 2.

**Table 2:** Results of a set of 1000 simulated sequences with different mutation rates.

	Number of being rejected^a^	IGHD		IGHD			IGHJ	
		
		Wrong pickups^b^	Accuracy (%)^c^	Missing^d^	Wrong pickups	Accuracy (%)	Wrong pickups	Accuracy (%)
Ab-origin	0	30	97.0	7	113	88.0	19	98.1
IMGT\V-QUEST	0	30	97.0	29	147	82.4*	44	95.6*
SoDA	0	87	91.3*	3	161	83.6*	83	91.7*
JOINSOLVER	26	161	81.3*	9	214	75.1*	46	92.8*
VDJsolver	97	80	82.3*	73	86	74.4*	35	86.8*
iHMMune-align	68	67	86.5*	22	111	79.9*	37	89.5*

The numbers represent the results from six programs. A wrong pickup means that the identified gene segment was not the one we used exactly in simulation.

^a ^Number of the rearranged sequences cannot be deciphered by the tool due to various reasons. Some tools give the explanation for rejection, i.e. JOINSOLVER could not well define the CDR3 region [15]; iHMMune-align is not intended for gaps in IGHV and short length of IGHV [17].

^b ^Number of the wrong pickups among the sequences already been deciphered.

^c ^Accuracy = 1-(wrong pickups+ number of missing+ number of being rejected)/1000

^d ^Number of the sequences already been deciphered by the tool but didn't find the IGHD alignment because of respective restrictions.

* Demonstrate that the accuracy of Ab-origin is significantly higher than that of the tool.

It can be seen from Table 2 that all the programs give higher accuracy in identifying IGHV and IGHJ gene segments than in identifying IGHD. This is because IGHD genes are much shorter and difficult to locate, as reported in previous studies [16,17]. Most of the wrong IGHV and IGHJ assignments are due to the existence of alleles. If factors such as mismatched alleles and the sequences rejected by the tool are excluded, IGHV and IGHJ can be identified respectively with accuracy close to 100% for the six tools.

In total, the performance of Ab-origin is the best among the 6 tools, with a statistically significant higher accuracy in classifying all the three types of gene segments (*p *< 0.05, Chi-square test, Table 2).

### Using the score from Ab-origin to estimate reliability of IGHD gene identification

In spite of the high accuracy when identifying IGHV and IGHJ, it is noticeable that identifying IGHD correctly is much more difficult for all the available tools. Without experimental results for reference, there is no criterion to ascertain which alignments are correct. Empirically, when the original D germline is not known, the consensus between all the tools is more likely to be the true result [17]. Unfortunately, further analysis of four non-redundant experimental datasets showed that the results from the five existing tools have only about 42% (average) agreement with each other in identifying IGHD gene segments (Additional File 2). This implies that the results from any one computational tool may contain a large number of false positives. Therefore for a specific tool, providing a scoring threshold to infer the confidence level would be desirable to users when the prediction results are obtained for IGHD. One possible way to derive scoring threshold is from a receiver operating characteristic (ROC) curve of large amount of simulated sequences.

**Figure 3:**
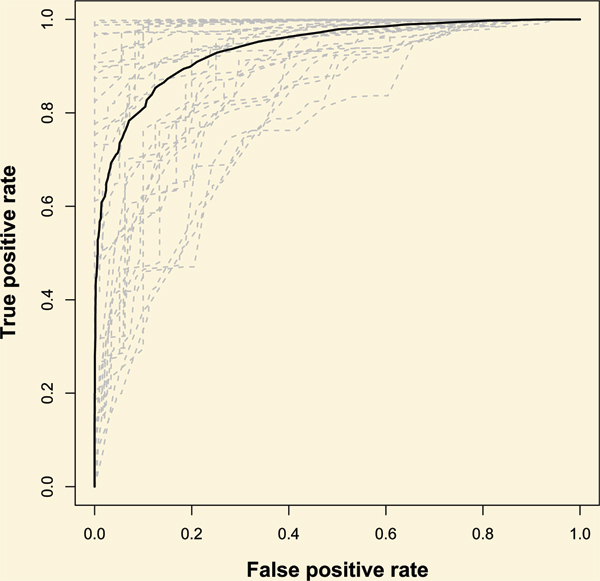
**ROC-curves for 32000 simulated sequences**. Solid line represents the ROC curve of IGHD identification derived from the 32000 sequences at gene level. There are 32 dashed lines (32 IGHD segments), each representing a ROC curve for the 1000 simulated sequences generated from one IGHD gene. X axis indicates (1-specificity); y axis indicates sensitivity.

An ROC curve is frequently adopted to evaluate the performance of a classifier [23], hence it is used in our study to examine whether our scores can successfully distinguish between right and wrong identifications. The ROC curve derived from IGHD identification results for 32000 simulated sequences of variable regions is plotted into Figure 3.

As a non-parametric measure of classification accuracy, ROC curve displays a trade-off between the sensitivity and specificity for all possible thresholds [24]. As in a case of random prediction, the true positive proportion would be equal to the false positive proportion for every threshold, and the ROC curve would be inclined to the diagonal. In other words, a good classifier would have a high true positive proportion as well as a low false positive proportion. Very much away from the diagonal, the solid line of the ROC curve in Figure 3 indicates that, the scoring from Ab-origin acts as a qualified classifier for the whole sequences set in general, although variances exist between sequences generated from different IGHD germline genes.

The relationship between selected thresholds and the sensitivity/specificity of the classification is summarized in Table 3. Users are strongly recommended to refer to this table for guidance when choosing the prediction results for further analysis.

**Table 3:** The relationship between selected threshold scores and the corresponding sensitivity/specificity

Threshold score	Sensitivity	Specificity
31	0.953	0.661
38	0.899	0.803
41	0.872	0.850
44	0.853	0.875
49	0.803	0.907
54	0.746	0.943
56	0.721	0.950

To examine whether our threshold is applicable to real data analysis, a threshold of 38 (with sensitivity = 0.9, specificity = 0.8) was applied as an example to the four sets of experimental antibody sequences which were analysed by the other five tools before (Additional File 2: Table S1). As shown Additional File 3: Table S2, the identified IGHD genes above this threshold have improved to 69% (average) agreement among the other five tools, in contrast to the 42% (average) agreement without any cut-off. Hence, such a threshold is expected to be a helpful guide to the credibility of the results. Since the respective accuracy for IGHV and IGHJ is high enough, their confidence were omitted.

## Discussion

The random assortment of the V, (D), J gene segments provides the basic structural frames for antibody variable region to recognize specific antigen. However, the details of this process are still largely unknown. Although the functional antibody sequences are plentiful, their origination V-D-J gene segments at germline level are seldom known. Till now only few experiments have studied the large-scale rearranged antibodies with known V gene source [18]. In this paper, experimental data of IGHV3-23 genes were first applied to examine the capability of different programs in picking up IGHV genes correctly, then to further derive the parameters of junctional flexibility for later simulation. Because of the scarcity of real data, validation of the performance of different algorithms has to heavily rely on simulated sequences in most cases. Hence artificial sequences were generated in our study for 32000 pieces of full-length variable regions of heavy chain. Though the simulation model is far from complete, it could be a practical way for evaluation purposes [16].

In recent years, several computational tools have emerged to identify the sourcing gene segments at germline level. After several rounds of validation, these tools showed different ability in finding V, D and J gene segments correctly for heavy chain. Generally speaking, programs based on sequence alignment gives better results in predicting longer segments, such as IGHV and IGHJ. However statistical models also show outstanding ability in tracing back D segment to its germline, such as iHMMune-align in Table 2, which indicates their future potential in computing short and volatile elements among the antibody sequence.

In spite of the various algorithms conceived, the accuracy for IGHD identification is lower than that of IGHJ and IGHV. There are several reasons which could contribute to the partitioning ability of algorithms: First is the length of the gene segments, it should be long enough in order to avoid random matches to its original germlines. Second is the number of gene segments in each of the V, D, J pools, the fewer the number of members in a group, the easier it is for the program to identify them. Third is the junctional flexibility and mutation rate. The prediction accuracy will be seriously affected if the gene segment varies too much during the rearrangement and further development process. The limited modification, fewer family segments, and sequences of sufficient length make IGHV and IGHJ segments to be identified with higher accuracy (Table 2), as demonstrated in previous studies [15,16,18]. With regard to IGHD identification, the germline sequences are much shorter than that of the IGHV and IGHJ, with average length of 24.35 ± 7.13 nt, and length ranges from 11 to 37 nt. In contrast, IGHV germline sequences vary from 288 to 305 nt and IGHJ from 48 to 63 nt. Furthermore, IGHD germline is the most volatile part which undergoes possible removal and N-addition from both ends, in addition to following somatic hypermutaion. Considering this, the remaining length of D segment in the rearranged sequences could be as short as few nucleotides from our statistical results (Additional File 1). Hence there is a higher probability of false positive matches in identifying D germlines, and in some cases, no hit can be found. That is partially explains why VDJsolver could not find the D segments if less than eight nucleotides long [18].

Ab-origin was developed based on BLAST, which has been widely accepted as a powerful and efficient algorithm for sequence alignment that allows customized parameter settings according to specific conditions [20,21]. In particular, for IGHD identification, Ab-origin applies a window-sliding strategy to exhaustively align the query sequences to the IGHD pool to find the best hit. Besides, the scoring scheme for IGHD search has been carefully evaluated and designed to minimize the influence of match length. It should be noted that Ab-origin is not suitable to compute cases of allelic exclusion, isotopic exclusion as a BLAST-based tool, however, with the accumulation of more functional antibody sequences, the abnormal features could be more evident and thus possible aberrant recombinations could be identified.

## Conclusion

An enhanced tool, Ab-origin, was developed to provide batch query services with joint advantages of accuracy and prediction confidence. Allowing detailed investigation of the original germline segment for antibodies and potential rearrangement profiles, Ab-origin is expected to serve as a useful tool for the informatics study in the immune-community, so as to promote the understanding of antibody maturation process. From current investigations, the most difficult part lies in the analysis of the junctional region, thus further efforts could be directed towards incorporating statistical models, such as HMM, and accumulating more experimental data to enable insightful research into the antibody rearrangement process.

## Methods

### Dataset

#### Germline data

Sequences of human IGHV, IGHD, IGHJ germline genes were retrieved from the IMGT reference directory (30/05/2008) [25]. 

#### Rearranged antibody sequences

Four sets of rearranged sequences of human immunoglobulin heavy chains have been prepared. Set one is 6329 clonally unrelated rearranged sequences which were collected from the testing data set of VDJsolver [18]. Other three sets were downloaded from the testing data of JOINSOLVER [15]. Set two consists of 404 sequences (Genbank accession numbers Z80363–Z80769); Set three consists of 120 sequences (Genbank accession numbers AY003749–AY003869); Set four consists of 143 sequences (Genbank accession numbers Z68345–Z68487).

### Searching algorithm of Ab-origin

V, D and J gene segments are assembled through a site-specific recombination reaction which is generally considered to be a random assortment [26]. To date, no evidence demonstrates that there is correlation between the use of V, D and J fragments during the recombination, thus V, D and J segments are searched separately when deciphering the rearranged sequences.

#### 1. V and J assignment

Firstly, BLAST algorithm is called to identify the best V gene segment from the database which shows the highest similarity to the query sequence of mature antibody gene. As the insertion/deletion events are infrequently found in the V gene segments [27], a rigorous penalty is set for gaps or extension of the gaps. Scoring system of +5 for match and -4 for mismatch is applied according to the suggestions from BLAST manual [21]. The word size is set to 7. Secondly, the best J segment is found with similar method mentioned above. Since J segment has a comparatively low point mutation rate, the penalty score is increased to -6 for mismatch.

Since the end site of IGHV and the start site of IGHJ can be located rather distinctly, V-to-J region is defined as the region between the IGHV end site to the IGHJ start site (Including N region between IGHV and IGHD, IGHD and N region between IGHD and IGHJ).

#### 2. D assignment

After the IGHV and IGHJ were identified respectively, the V-to-J region was compared to all IGHD germlines in the database. BLAST algorithm is applied to identify the best D gene segment, where the match length of the alignment should be no less than 60% of the total length of individual D segment or V-to-J region length to avoid local alignment. In other cases, the IGHD gene was aligned to the V-to-J region by a sliding window at step-size of one nucleotide. Five nucleotide protrusions in D segment are allowed at both ends during the alignment considering the junctional flexibility. An optimized score scheme of +5/-4 (+5 for match, -4 for mismatch) was chosen in this alignment based on the simulation process described below. Only the alignment scores above certain threshold (see "Score threshold for non-random match" described below) were recorded to find the best match.

### Monte Carlo simulation of V-to-J region to find optimized scoring scheme

In order to evaluate how the different segment length affects the scoring scheme, 1000 sequences for each length from 5 to 64 nt long were randomly generated according to the length distribution of V-to-J region. Varied scoring scheme of +5/-x(x from zero to ten, stepping one) was applied to the alignment between the randomized V-to-J sequences and D germline database. Score coefficient of variations for sequences of various lengths were calculated and plotted according to different X value.

### Score threshold for non-random match

For a given V-to-J region of length m (from 5 to 64 nt), a score threshold was needed to identify a D gene significantly instead of a random match. 1000 sequences of length *m *were randomly generated similarly and the scores of alignment by the above optimized scoring scheme were recorded. The threshold was set to the 95% quantile of the sorted scores, corresponding to a *p*-value of 0.05. Only scores above these thresholds will be considered as significant match.

### Simulation of variable regions for heavy chain to evaluate the accuracy of Ab-origin

For each IGHD gene, a set of 1000 rearranged sequences were generated by randomly selecting IGHV and IGHJ genes.

The next step is to introduce the junctional flexibility, including exonuclease removals and insertion of N-region, into the V-D and D-J joint region of the simulated sequences. In this study, 0 to 5 nt were randomly cut off from the 3' end of V gene, the 5'end of J gene and the both ends of D gene, according to a previous research [22]. Then, up to 15 N-nucleotides were randomly added in the simulation of the D-J and V-D joining.

The last step is to introduce point mutations randomly and independently for the simulated V-D-J sequences, taking into account that the transition rate is twice as much as the transversion rate in the somatic hypermutation [7]. Mutation rates, ranging from 0% to 15% stepping 1%, was randomly set for each sequence to simulate the different phase of antibody affinity maturation.

In total we got 32000 sequences. The flowchart of simulation is presented in Additional File 4: Figure S2.

### ROC curve

ROCR package was adopted here for ROC calculation to test Ab-origin based on IGHD results [28]. For the total set of 32000 simulated sequences, target value is set to be one when the IGHD gene was correctly picked up and zero otherwise. Every IGHD identification can be classified as positives or negatives according to different score threshold. While according to the target values, the predictions can be true or false.

The IGHD assignment can be categorized as true positives (TP), true negatives (TN), false positives (FP) or false negatives (FN). For every value of the score threshold, the true positive rate, TP/(TP+FN), and the false positive rate, FP/(FP+TN), is calculated respectively. The sensitivity equals to the true positive proportion, and the specificity, given by TN/(FP+TN), equals (1 – the false positive proportion). A ROC curve is constructed by plotting the sensitivity against the specificity for all values of the threshold.

### Implementation

Ab-origin was developed using the Java language and is therefore platform independent. Currently a compiled version (which does not require java environment) is available for downloading at  along with simulation data used in this study.

## List of abbreviations used

IGHV: immunoglobulin heavy chain variable gene segment; IGHJ: immunoglobulin heavy chain joining gene segment; IGHD: immunoglobulin heavy chain diversity gene segment; nt: nucleotides; CDR: complementary determine region; Ig: immunoglobulin.

## Competing interests

The authors declare that they have no competing interests.

## Authors' contributions

XW designed and implemented the method, evaluated the results and drafted the manuscript. DW contributed to statistic analysis of the results. SZ conducted the simulations. JS and LT contributed to retrieve the result from other five tools. YXL and ZWC contributed to conceive of the study and revise the manuscript. All authors read and approved the final manuscript.

## Supplementary Material

Additional file 1 **Figure S1: Length distribution of V-to-J region for simulated and real sequences**. The figure demonstrates that length distribution of the V-to-J region of 32000 simulated sequences has no significant difference from that of 4450 real antibody sequences. Click here for file

Additional file 2 **Table S1: Results of IGHD identification among five existing tools from four sets of antibody heavy chain sequences**. The agreements of these five programs (IMGT/V-QUEST, SoDA, JOINSOLVER, VDJsolver and iHMMune-align) in IGHD identification at the allele level. Click here for file

Additional file 3 **Table S2: Results of IGHD identification from Ab-origin with scores higher than 38**. The agreement between Ab-origin to five programs (IMGT/V-QUEST, SoDA, JOINSOLVER, VDJsolver and iHMMune-align) in IGHD identification with score >= 38 at the allele level. Click here for file

Additional file 4 **Figure S2: Simulation flowchart of antibody maturation process**. The figure demonstrates the flowchart for the simulation of antibody maturation in our study. Each IGHD simulated 1000 times independently. Click here for file
