# Supplementary figures and images for: Ab-origin: an enhanced tool to identify the sourcing gene segments in germline for rearranged antibodies
Source: BMC Bioinformatics. 2008 Dec 12;9(Suppl 12):S20. doi: 10.1186/1471-2105-9-S12-S20 (PMC2638160; doi:10.1186/1471-2105-9-S12-S20)

V-to-J region length distribution

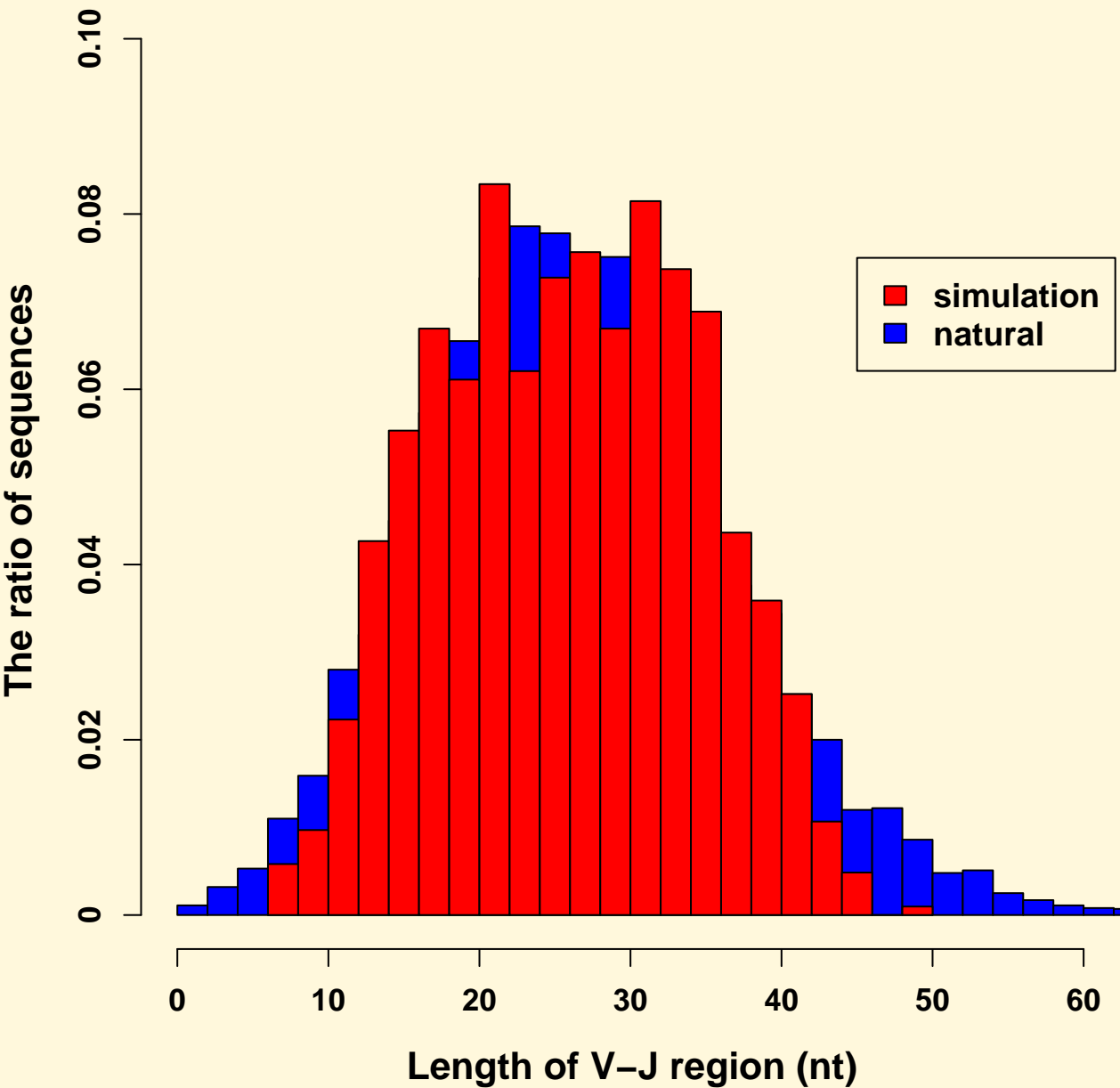

Supplement: Additional file 1 — Figure S1: Length distribution of V-to-J region for simulated and real sequences. The figure demonstrates that length distribution of the V-to-J region of 32000 simulated sequences has no significant difference from that of 4450 real antibody sequences. [file 1471-2105-9-S12-S20-S1.pdf]

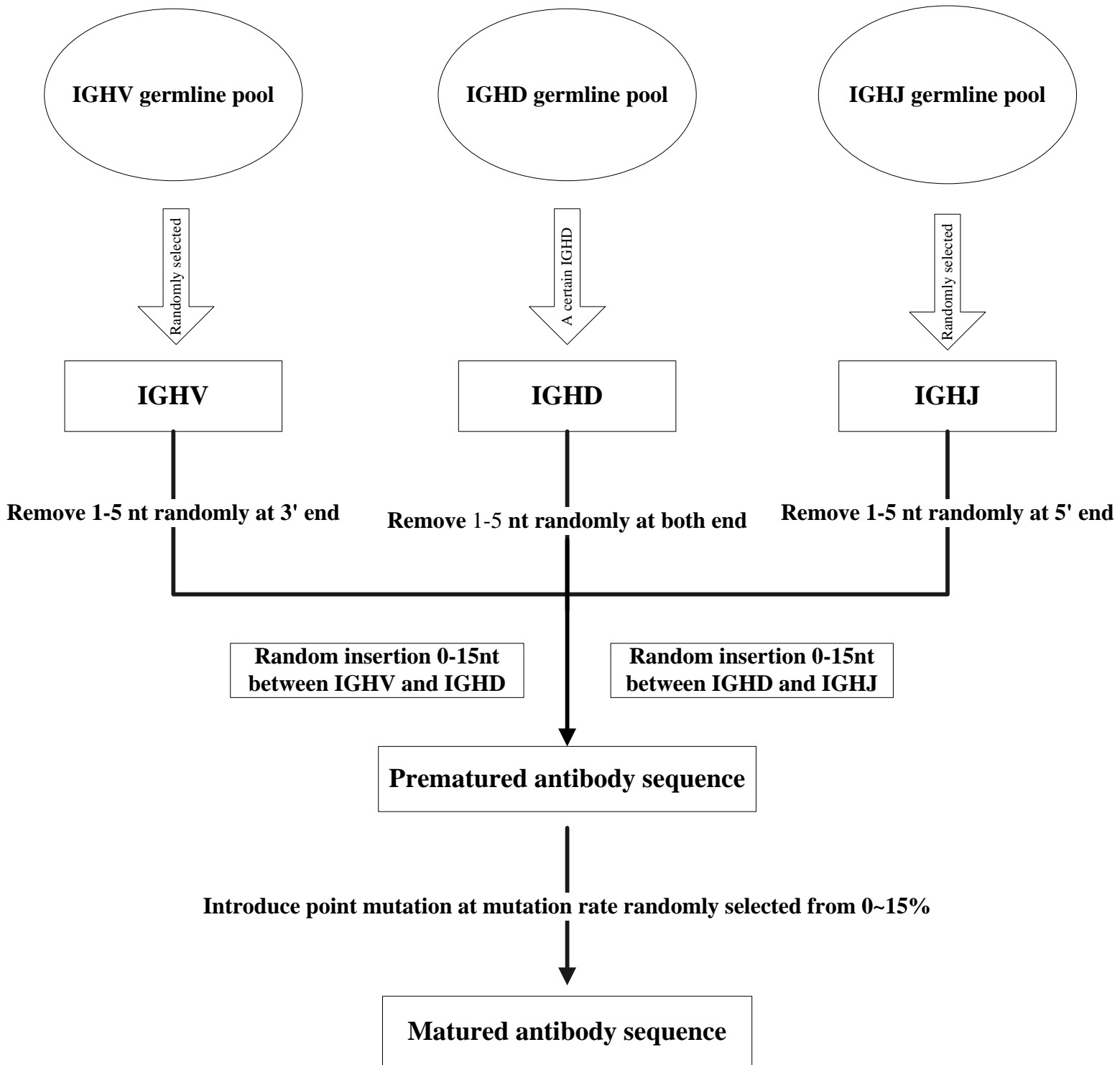

Supplement: Additional file 4 — Figure S2: Simulation flowchart of antibody maturation process. The figure demonstrates the flowchart for the simulation of antibody maturation in our study. Each IGHD simulated 1000 times independently. [file 1471-2105-9-S12-S20-S4.pdf]
