# Supplementary material for: Ab-origin: an enhanced tool to identify the sourcing gene segments in germline for rearranged antibodies
Source: BMC Bioinformatics. 2008 Dec 12;9(Suppl 12):S20. doi: 10.1186/1471-2105-9-S12-S20 (PMC2638160; doi:10.1186/1471-2105-9-S12-S20)
Supplement: Additional file 2 — Table S1: Results of IGHD identification among five existing tools from four sets of antibody heavy chain sequences. The agreements of these five programs (IMGT/V-QUEST, SoDA, JOINSOLVER, VDJsolver and iHMMune-align) in IGHD identification at the allele level. [file 1471-2105-9-S12-S20-S2.doc]

### Table S1: Agreement among five existing tools in identifying IGHD gene segment from four sets of antibody heavy chain sequences

The agreements of these five programs (IMGT/V-QUEST, SoDA, JOINSOLVER, VDJsolver and iHMMune-align) in IGHD identification at the allele level.

|  | Total Number | Agreement with all 5 tools | | Agreement with any 4 tools | | Agreement with any 3 tools | | Data Resource |
| --- | --- | --- | --- | --- | --- | --- | --- | --- |
|  |  | number | ratio | number | ratio | number | ratio |  |
| set1 | 500 | 230 | 0.46 | 302 | 0.60 | 357 | 0.71 | [1] |
| set2 | 404 | 173 | 0.43 | 237 | 0.59 | 287 | 0.71 | [2] |
| set3 | 120 | 50 | 0.42 | 70 | 0.58 | 90 | 0.75 | [2] |
| set4 | 143 | 54 | 0.38 | 73 | 0.51 | 91 | 0.64 | [2] |
| average | 292 | 127 | 0.42 | 171 | 0.57 | 206 | 0.70 | - |

1. L Ohm-Laursen, M Nielsen, SR Larsen, T Barington: **No evidence for the use of DIR, D-D fusions, chromosome 15 open reading frames or VH replacement in the peripheral repertoire was found on application of an improved algorithm, JointML, to 6329 human immunoglobulin H rearrangements**. *Immunology* 2006, **119**:265-77.

2. MM Souto-Carneiro, NS Longo, DE Russ, HW Sun, PE Lipsky: **Characterization of the human Ig heavy chain antigen binding complementarity determining region 3 using a newly developed software algorithm, JOINSOLVER**. *J Immunol* 2004, **172**:6790-802.
