# Supplementary material for: Ab-origin: an enhanced tool to identify the sourcing gene segments in germline for rearranged antibodies
Source: BMC Bioinformatics. 2008 Dec 12;9(Suppl 12):S20. doi: 10.1186/1471-2105-9-S12-S20 (PMC2638160; doi:10.1186/1471-2105-9-S12-S20)
Supplement: Additional file 3 — Table S2: Results of IGHD identification from Ab-origin with scores higher than 38. The agreement between Ab-origin to five programs (IMGT/V-QUEST, SoDA, JOINSOLVER, VDJsolver and iHMMune-align) in IGHD identification with score >= 38 at the allele level. [file 1471-2105-9-S12-S20-S3.doc]

### Table S2: Results of IGHD identification from Ab-origin with scores higher than 38.

The agreement between Ab-origin to five programs (IMGT/V-QUEST, SoDA, JOINSOLVER, VDJsolver and iHMMune-align) in IGHD identification with score >= 38 at the allele level.

|  | Total Number | Score >=38^a^ | Agreement with | | | | | No  agree-  ment |
| --- | --- | --- | --- | --- | --- | --- | --- | --- |
|  |  |  | all 5 ^b^ | any 4 | any 3 | any 2 | only 1 |  |
| set1 | 500 | 252 | 0.74 | 0.83 | 0.88 | 0.91 | 0.96 | 0.04 |
| set2 | 404 | 231 | 0.68 | 0.81 | 0.86 | 0.89 | 0.93 | 0.07 |
| set3 | 120 | 66 | 0.65 | 0.79 | 0.85 | 0.89 | 0.95 | 0.05 |
| set4 | 143 | 68 | 0.69 | 0.81 | 0.87 | 0.90 | 0.96 | 0.04 |
| average | 292 | 154 | 0.69 | 0.81 | 0.86 | 0.90 | 0.95 | 0.05 |

^a^ The number of IGHD identification agree with all other five tools with score>=38

^b^$\text{Agreement ratio = }\frac{\text{Number of IGHD identifications that agree with all the five tools}}{\text{Number of IGHD identifications with score>=38}}$The rest can be deduced by analogy.
